# Neuronal Splicing Regulator RBFOX3 (NeuN) Regulates Adult Hippocampal Neurogenesis and Synaptogenesis

**DOI:** 10.1371/journal.pone.0164164

**Published:** 2016-10-04

**Authors:** Yi-Sian Lin, Han-Ying Wang, De-Fong Huang, Pei-Fen Hsieh, Meng-Ying Lin, Chih-Hsuan Chou, I-Ju Wu, Guo-Jen Huang, Susan Shur-Fen Gau, Hsien-Sung Huang

**Affiliations:** 1 Graduate Institute of Brain and Mind Sciences, College of Medicine, National Taiwan University, Taipei, Taiwan; 2 Department of Biomedical Sciences, Chang Gung University, Tao-Yuan, Taiwan; 3 Department of Psychiatry, College of Medicine, National Taiwan University, Taipei, Taiwan; 4 Clinical Center for Neuroscience and Behavior, National Taiwan University Hospital, Taipei, Taiwan; 5 Neurobiology and Cognitive Science Center, National Taiwan University, Taipei, Taiwan; 6 Ph.D. Program in Translational Medicine, National Taiwan University and Academia Sinica, Taipei, Taiwan; 7 Neurodevelopment Club in Taiwan, Taipei, Taiwan; University of South Florida, UNITED STATES

## Abstract

Dysfunction of RBFOX3 has been identified in neurodevelopmental disorders such as autism spectrum disorder, cognitive impairments and epilepsy and a causal relationship with these diseases has been previously demonstrated with *Rbfox3* homozygous knockout mice. Despite the importance of RBFOX3 during neurodevelopment, the function of RBFOX3 regarding neurogenesis and synaptogenesis remains unclear. To address this critical question, we profiled the developmental expression pattern of *Rbfox3* in the brain of wild-type mice and analyzed brain volume, disease-relevant behaviors, neurogenesis, synaptic plasticity, and synaptogenesis in *Rbfox3* homozygous knockout mice and their corresponding wild-type counterparts. Here we report that expression of *Rbfox3* differs developmentally for distinct brain regions. Moreover, *Rbfox3* homozygous knockout mice exhibited cold hyperalgesia and impaired cognitive abilities. Focusing on hippocampal phenotypes, we found *Rbfox3* homozygous knockout mice displayed deficits in neurogenesis, which was correlated with cognitive impairments. Furthermore, RBFOX3 regulates the exons of genes with synapse-related function. Synaptic plasticity and density, which are related to cognitive behaviors, were altered in the hippocampal dentate gyrus of *Rbfox3* homozygous knockout mice; synaptic plasticity decreased and the density of synapses increased. Taken together, our results demonstrate the important role of RBFOX3 during neural development and maturation. In addition, abnormalities in synaptic structure and function occur in *Rbfox3* homozygous knockout mice. Our findings may offer mechanistic explanations for human brain diseases associated with dysfunctional RBFOX3.

## Introduction

RBFOX3/NeuN belongs to the RNA-binding Fox (Rbfox) family of proteins and the whole family of proteins regulate alternative RNA splicing [1]. *Rbfox3* is exclusively expressed in neurons and dysfunctional RBFOX3 has been observed in various human brain disorders such as epilepsy [2], autism spectrum disorder [3], neurodevelopmental delay [3, 4], and cognitive impairments [5]. We previously used *Rbfox3* homozygous knockout mice to demonstrate a causal relationship between RBFOX3 and these diseases [6]. However, the function of RBFOX3 in the brain remains poorly understood. A recent study suggests RBFOX3 regulates neuronal differentiation by alternative splicing of *Numb* pre-mRNA [7] and our recent finding showed RBFOX3 is required for hippocampal circuit balance and function [6]. These two findings indicate RBFOX3 is critical for brain development but it is unclear how *Rbfox3* is developmentally regulated and whether RBFOX3 could contribute to normal brain and synaptic structure.

Cognitive deficits identified in persons with dysfunctional RBFOX3 [5] are similar to spatial learning deficits identified in *Rbfox3* homozygous knockout mice [6]. Evidence also suggests the hippocampal dentate gyrus (DG) may also play a role in cognition, through adult neurogenesis, which affects learning and memory [8, 9] and adult synaptogenesis, which affects learning behaviors [10, 11]. The spatial learning deficits exhibited by *Rbfox3* homozygous knockout mice and persons with dysfunctional RBFOX3 suggest RBFOX3 may play a role in adult neurogenesis and synaptogenesis.

To investigate the role of RBFOX3 during brain development with emphasis on neurogenesis and synaptogenesis, we first profiled the developmental expression pattern of *Rbfox3* in wild-type mice. We then used *Rbfox3* homozygous knockout (*Rbfox3*^*−/−*^) mice as an animal model to determine the effects of RBFOX3 on brain volume, disease-relevant behaviors, neurogenesis, synaptic plasticity and synaptogenesis. We found that expression of *Rbfox3* differs developmentally in distinct brain regions. In addition, *Rbfox3*^*−/−*^ mice displayed cold hyperalgesia and impaired cognition. Consistent with hippocampal DG circuit dysfunction, *Rbfox3*^*−/−*^ mice had defective synaptic plasticity and density in the hippocampal DG. Moreover, RBFOX3 regulates the exons of genes with synapse-related function. Our findings indicate the potential contributions of RBFOX3 to the pathogenesis of brain diseases with disrupted *RBFOX3*.

## Materials and Methods

### Mice

*Rbfox3*^*−/−*^ mice (full strain nomenclature is C57BL/6N-*Rbfox3*^*tm1a(EUCOMM)Hmgu*^, EM: 04705) were purchased from the Mary Lyon Centre of MRC Harwell (Oxford, UK); detailed information about these mice can be obtained in our previous study [6]. Mice were group-housed in ventilated cages, given food (PicoLab^®^ Rodent Diet 20, 5053) and water *ad libitum* and maintained on a 12-h light/dark cycle (lights off at 8 pm). Male *Rbfox3* heterozygous knockout mice were mated to female *Rbfox3* heterozygous knockout mice to obtain *Rbfox3* homozygous knockout mice and littermate control wild-type mice. The National Taiwan University College of Medicine and the College of Public Health Institutional Animal Care and Use Committee (IACUC) approved all procedures in this study. All experiments in this study were performed in accordance with the approved guidelines.

### Western blotting

Tissues were isolated and extracted from different brain regions and developmental stages. The brain hemispheres were taken randomly. Protein extracts were obtained by homogenizing tissues in lysis buffer (1% Triton X-100, 5 mM EDTA, pH 8, 0.15 M NaCl, 10 mM Tris-HCl, pH 7.5, Halt^™^ Protease and Phosphatase Inhibitor Cocktail (1:100, Thermo Scientific, 78440)) and protein concentration was determined with the Pierce BCA Protein Assay Kit (Thermo Scientific, 23227). Total protein lysates (40 μg) were separated by 7.5% SDS-polyacrylamide gel electrophoresis and transferred to nitrocellulose membranes. Immunoblotting was performed using mouse anti-NeuN (1:1,000, Millipore, MAB377) and mouse anti-beta-actin (1:5,000, Sigma, A1978) primary antibodies. Primary antibodies were detected with their corresponding secondary antibody: IRDye680 donkey anti-mouse IgG (H+L) (1:15,000, LI-COR, 926–68072). Protein bands were visualized using an Odyssey^®^ Fc Dual-Mode Imaging System (LI-COR Biosciences). The intensities of protein bands were analyzed using the Image Studio^™^ software (LI-COR Biosciences, version 4.0.21). To control for protein loading, each protein level was normalized to beta-actin levels detected in each sample.

### Magnetic Resonance Imaging (MRI) volumetric analysis

Adult male mice (P56) were deeply anesthetized with isoflurane and perfused transcardially with 0.1M phosphate buffer (PB) with heparin (30 U/ml Heparin, Sigma, H-4784) followed by 4% paraformaldehyde in 0.1 M PB, pH 7.4. Mouse brains were removed, post-fixed overnight, and cryoprotected with 30% sucrose in 0.1 M PB for two days. We determined the volumes of the whole brain and different brain regions with MRI volumetry using a Biospec 4.7T 40-cm bore horizontal MRI system. The FSE T2WI parameters are below. TR = 6000 ms, TE = 60 ms, NEX = 10, FOV = 2x2 cm, slice-thickness = 0.5 mm, matrix size = 256x256, scanning time = 32 min.

### Behavioral measures

All behavioral assessments were performed with two cohorts of WT and *Rbfox3*^*-/-*^ male mice. The first cohort of mice (12 WT mice and 9 *Rbfox3*^*-/-*^ mice) was derived from 14 litters and used for gait analysis, grip force test, and prepulse inhibition test. The second cohort (8 WT mice and 8 *Rbfox3*^*-/-*^ mice) was derived from 9 litters and used for the rotarod test, hot/cold plate test and novel object recognition test. Testing began when mice were 7–11 weeks of age (1^st^ cohort) and 8–13 weeks of age (2^nd^ cohort). Mice were sacrificed immediately at the end of behavioral measures and were not further used for any biochemical measures in this study.

#### Rotarod test

Mice were evaluated for balance and motor coordination on an accelerating rotarod (Ugo Basile). Revolutions per minute (rpm) were set at an initial value of 3 rpm, with a progressive increase to a maximum of 30 rpm across 5 min, the maximum trial length. Test sessions consisted of 3 trials, with 1 min between each trial. Latency to fall, or to rotate off the top of the turning barrel, was determined by the rotarod timer.

#### Gait analysis

Mice were placed individually in an enclosed Catwalk walkway, which consisted of a glass plate plus two Plexiglas walls. The mice were allowed to walk freely and traverse from one side to the other of the walkway glass plate. Two infrared light beams were used to detect the arrival of the mouse and to control the start and end of data acquisition. The recordings were carried out when the room was completely dark. During the experiment, the crossing of the mice was captured by a high-speed video camera. The footprints and gait were automatically detected in an enclosed walkway with the CatWalk gait analysis system (Noldus Information Technology) and analyzed by CatWalk XT software. Paw pressure (light intensity), paw print area (complete surface area contacted by the paw during a stance phase) and interlimb coordination (measured as regularity index, which is the percentage of normal step sequences) were analyzed.

#### Grip force test

Grip-strength of forelimbs was measured by the Grip force test system (UGO Basile). When pulled by the tail, the mice instinctively grasp anything to stop backward movement. Mice were placed over a base plate before a grasping bar. The bar was fitted to a force transducer connected to the peak amplifier. Using this device, mice were pulled by the tail and maximal grip force (g) was measured within 20 seconds. Test sessions consisted of 3 trials, with 10 min between each trial.

#### Hot/cold plate test

Hot/cold plate test measures thermal nociception. The hot plate apparatus (Ugo Basile) was maintained at 55.0±1°C. Mice were placed into a glass cylinder of 20 cm diameter on the heated surface, and the time between placing of the mouse on the hot plate and the occurrence of licking of hind paws or jumping off the surface was recorded as response latency (s). A 50 s cut-off was used to prevent tissue damage. The cold plate test used the same instrument and procedures as the hot plate apparatus (Ugo Basile), however the surface was maintained at 0.0±1°C.

#### Prepulse inhibition measurements

Prepulse inhibition (PPI) was determined with the acoustic startle measure, which is based on the reflexive whole-body flinch, or startle response, following exposure to a sudden noise. Mice were tested with the Startle reflex test system (San Diego Instruments SR-LAB). Briefly, mice were placed in a small Plexiglas cylinder within a larger, sound-attenuating chamber (San Diego Instruments). The cylinder was seated upon a piezoelectric transducer, which allowed vibrations to be quantified and displayed on a computer. The chamber included a houselight, fan, and a loudspeaker for the acoustic stimuli (bursts of white noise). Background noise levels (70 dB) and calibration of the acoustic stimuli were confirmed with a digital sound level meter (San Diego Instruments). Each test session consisted of 56 trials, presented following a 4-min habituation period. There were four different types of trials: no-stimulus trials, trials with the acoustic startle stimulus (40 ms; 115 dB) alone, and trials in which a prepulse stimulus (20 ms; either 77 or 86 dB) had onset 100 ms before the onset of the startle stimulus. The different trial types were presented in blocks of four, in randomized order within each block, with an average inter-trial interval of 15 sec (range: 10 to 20 sec). Measures were taken of the startle amplitude for each trial, defined as the peak response during a 65-msec sampling window that began with the onset of the startle stimulus. The percentage PPI of the startle response was calculated as 100 –[(SRPP/SR) x 100], where SR means the startle response to the pulse stimulus and SRPP means the startle response to the pulse with the prepulse stimulus.

#### Novel object recognition test

The novel object recognition test measures recognition memory. The test was carried out in an open field box measuring 47 cm x 26 cm x 21 cm. Mice were habituated to the test box for 10 min for the first 2 days with no objects present in the environment (the habituation phase). On the third day, mice were placed in the test box and after a 10-min habituation period, two identical objects were introduced in two corners (approximately 20 cm apart from each other). The objects used in this study were 4 cm x 4 cm x 4 cm plastic blocks (the familiarization phase). The total time spent exploring each object was recorded using a 20s criterion for exploration time. Then the mice were returned to their home cage. After 24-hr the mice were placed in the test box once again and the test phase began. The test box contained one of the familiar objects used in the previous training session and a novel object. Since mice showed no preference for particular objects or locations, the novel objects were introduced on the right side of the test box. The time spent exploring the familiar and the novel object during the testing phase was represented as *a*, and *b*, respectively. The discrimination index was calculated as (*b*-*a*)/(*a*+*b*). Two different cohorts were conducted for the novel object recognition test. We normalized the discrimination index for each cohort of data before combining the two cohorts.

#### Neurogenesis analysis

Adult mice (P80) were deeply anesthetized with isoflurane and perfused transcardially with 0.1M PB, pH 7.4 with heparin (30 U/ml Heparin, Sigma, H-4784) followed by 4% paraformaldehyde in 0.1 M PB, pH 7.4. Mouse brains were removed, post-fixed overnight, and cryoprotected with 30% sucrose in 0.1 M PB for two days. Coronal sections of the hippocampus were cut at a thickness of 40 μm on a sliding microtome. Sections were mounted on SuperFrost slides and dried overnight. Subsequently, slides were incubated in 0.01 mol/L citrate buffer for 40 min at 90°C, 3% H_2_O_2_ for 10 min, rinsed in PBS, and incubated overnight at room temperature in a goat polyclonal antibody to DCX (1:400, Santa Cruz, sc-8066) or a mouse monoclonal antibody to Ki67 (1:3000, Vector Lab, VP-K451). The following day, sections were stained with biotinylated secondary antibodies (standard IgG ABC kit, Vector Lab, PK-6105 for DCX; PK6101 for Ki67) followed by incubation for 5–10 min in 3’3’-diaminobenzidene (Sigma). Sections were then counterstained with cresyl violet and mounted with DPX (Sigma, 06522). Images were acquired using a Zeiss Axio Imager M2 microscope with a 10X/0.3 NA objective. DCX- and Ki-67-positive cells were counted in every eighth section throughout the entire rostrocaudal extent of the DG hemilaterally (6 sections per mouse brain). The DCX- and Ki-67-positive cells in all focal planes through the 40-μm section were included. The total number of labelled cells per section was determined and multiplied by section selection ratio (eight) and the number of DG per mouse brain (two) to obtain the total number of labelled cells in the DG.

### Hippocampal slice preparation

Mice (P48 to P51) were anaesthetized with 5% isoflurane in oxygen-enriched air and decapitated. Brains were rapidly removed and chilled in ice-cold dissection buffer containing (in mM): 87 NaCl, 2.5 KCl, 0.5 CaCl_2_, 7 MgCl_2_, 1.25 NaH_2_PO_4_, 25 NaHCO_3_, 75 sucrose, 10 glucose and 1.3 ascorbic acid, oxygenated with 95% O_2_ and 5% CO_2_ (pH, 7.4; 300 mOsmol). Hippocampal slices were cut coronally at 350 μm in dissection buffer using a vibroslicer (VT 1200S, Leica, Buffalo Grove, IL). Prior to recording, slices were allowed to recover by incubating for 30 min in 30°C artificial cerebrospinal fluid (ACSF) consisting of (mM): 124 NaCl, 3 KCl, 2 CaCl_2_, 1 MgCl_2_, 1.25 NaH_2_PO_4_, 26 NaHCO_3_ and 20 glucose (pH, 7.4; 295 mOsmol) and gassed with 95% O_2_ and 5% CO_2_. And then maintaining in a moist air-liquid (ACSF) interface chamber at room temperature for 60 min. Individual slices were transferred to an immersion-type recording chamber mounted on an upright microscope (Axio Examiner D1, Zeiss) and continuously perfused with oxygenated ACSF at a rate of 2–3 ml/min and maintained at 30–32°C. Neurons were viewed using Nomarski optics.

### Field potential recordings

Field excitatory postsynaptic potentials (fEPSPs) were recorded in coronal hippocampal slices with borosilicate glass electrodes (1.5-mm outer diameter, 0.32-mm wall thickness; Sutter, Novato, CA, USA) with a resistance of approximately 2–3 MΩ when filled with ACSF. Both recording electrode and bipolar stainless steel electrode (CBCMX75 (ST1)), FHC, Inc., Bowdoinham, ME, USA) were posed in the medial perforant path of the molecular layer. fEPSPs were recorded in the presence of 5 μM SR95531 (Abcam, ab120042), 3μM CGP54626 (Tocris, 1088) and 1μM Strychnine (Abcam, ab120416) and were evoked every 20 s. In each experiment, an input-output (I-O) relationship was plotted by the initial slope of the fEPSP versus the fiber volley. Stimulus intensity was adjusted to about 30% amplitude of the maximum fEPSPs. Paired stimuli were delivered between 50, 100, 200, 500 and 1000 ms apart and paired-pulse ratios (PPR) were measured by dividing the initial slope of the second fEPSP by the initial slope of the first fEPSP. For chemical induced LTD (chemical-LTD) recoding, 50 ms paired stimuli were delivered for inducing fEPSP and monitoring PPR. 30 μM DHPG (Tocris, 0342) was added into bath solution for 20 minute to induce chemical-LTD following 20-min stable baseline recording. In rescue experiments, 100 nM bryostatin-1 (Tocris, 2383) or vehicle was applied in whole recording (from 20 min baseline to 60 min chemical-LTD). Changes in synaptic strength were measured by comparing the average response slopes 45–60 min after conditioning stimulation to the pre-conditioning baseline response (5–20 min).

### RNA-sequencing (RNA-Seq)

Mice were deeply anesthetized with isoflurane before being sacrificed. The right cerebral cortical hemisphere was removed from P56 male WT and *Rbfox3*^*-/-*^ mice. Total RNA was extracted with the RNeasy lipid tissue mini kit (Qiagen, 74804). The details of RNA quality check, cDNA library construction, and differential gene expression analysis have been previously described [6]. For exon usage analysis, DEXSeq, a statistical method to test for differential exon usage in RNA-Seq data [12], was applied in this study. We aligned the RNA-Seq reads from WT and KO samples to the mouse genome (Ensembl 69) using BWA. After alignment, we selected the exons with 10 reads mapped to further DEXSeq analysis, and a false discovery rate of 1% was set to find significant differential exon usage. For binding pattern recognition, we fetched seq of the 500 bp upstream and downstream of each exon of the mouse ref genome (Ensembl 69) to find the TGCATG pattern in these sequences. Gene ontology (GO) analysis was performed with the GOrilla program (http://cbl-gorilla.cs.technion.ac.il/) [13, 14]. The RNA-Seq data discussed in this publication have been deposited in NCBI’s Gene Expression Omnibus [15] and are accessible through GEO Series accession number GSE84786 (http://www.ncbi.nlm.nih.gov/geo/query/acc.cgi?acc=GSE84786).

### Synaptic number and size analysis

Adult mice (P49) were deeply anesthetized with isoflurane and perfused transcardially with 0.1M PB with heparin (30 U/ml Heparin, Sigma, H-4784) followed by with 4% paraformaldehyde in 0.1 M PB, pH 7.4. Mouse brains removed, post-fixed overnight, and cryoprotected with 30% sucrose in 0.1 M PB for two days. Coronal dorsal hippocampal sections (7 μm) were cut on a cryostat (Leica, CM3050 S), and every eleventh section was mounted on glass slides. Sections were permeabilized with 0.3% Triton X-100 in 1X PBS for 30 min and blocked with 5% goat serum for 2 hr. Sections were incubated with primary antibodies (rabbit anti-Synapsin 1 (SYN1), 1:1,000, Millipore, AB1543; mouse anti-PSD95, 1:200, Thermo Fisher, 7E3-1B8) at 4°C overnight followed by 2 hr incubation at room temperature with Alexa Fluor^®^ 488 Goat anti-mouse IgG1 (γ1) secondary antibody (1:500, Life Technologies, A21121), Alexa Fluor^®^ 546 Goat anti-rabbit IgG (H+L) secondary antibody (1:500, Life Technologies, A11010) and DAPI (1,1,000, Invitrogen, D-1306). Images for synaptic counting were acquired using a Zeiss LSM880 confocal microscope (Carl Zeiss, Taiwan) with a 100X/1.46 NA objective. The density and size of puncta or synapses of proximal dendrites (100 μm from the soma) of granule cells in the DG was quantified via ImageJ and Synapse Counter (plug-in for ImageJ, https://github.com/SynPuCo/SynapseCounter) through a depth 1 μm-thick section to optimize visualization of definable puncta. Schematic images were acquired using a Zeiss Axio Imager M2 microscope with a 10X/0.3 NA objective and a 40X/0.75 NA objective (enlarged images).

### Statistical analysis

All data are presented as means ± standard error of the mean (s.e.m.) with sample sizes (n) shown in figures or stated in the text. Statistical analyses (Student’s t-test, One-way ANOVA and Two-way repeated measures ANOVA) were performed using SigmaPlot 13.0 (Systat Software). Normality tests (Shapiro-Wilk) and equal variance tests were run and passed (*P* > 0.05) before parametric statistical analyses were performed. *Post hoc* analyses (Holm-Sidak and Tukey) were performed if needed. Non-parametric statistical analyses (Kruskal-Wallis One-way ANOVA on ranks and Mann-Whitney Rank Sum Test) were performed if normality and equal variance tests were not passed (*P* < 0.05).

## Results

### Expression of *Rbfox3* differs developmentally in distinct brain regions

**Fig 1:**
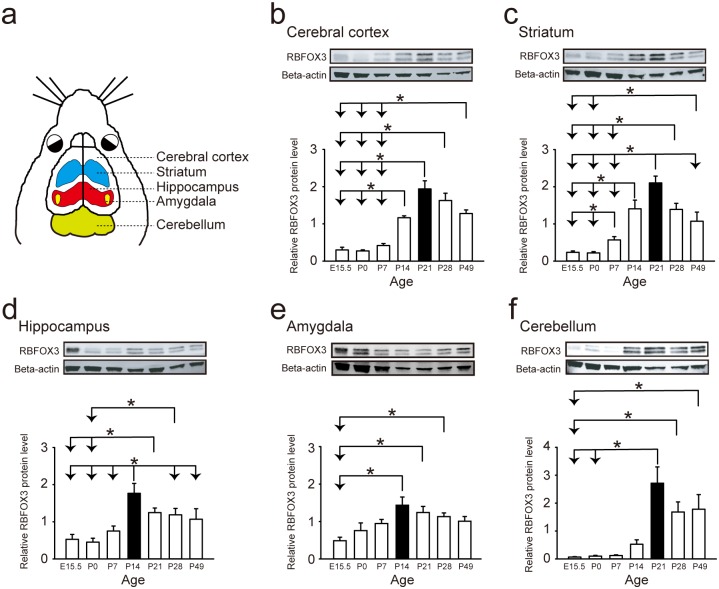
*Rbfox3* is developmentally expressed and regulated in distinct brain regions. (**a**) Schematic of brain regions examined in WT mice. Levels of RBFOX3 protein were measured from embryonic stage (embryonic day 15.5, E15.5) to adult stage (postnatal day 49, P49) in the cerebral cortex (**b**), striatum (**c**), hippocampus (**d**), amygdala (**e**) and cerebellum (**f**) with western blot analysis. One-way ANOVA with Holm-Sidak *post hoc* comparison, *P < 0.05 for (**b-e**), Kruskal-Wallis One-way ANOVA on ranks with Tukey *post hoc* comparison, *P < 0.05 for (**f**). n = 5 brain hemispheres. All data are the mean ± s.e.m.

Neurodevelopmental impairments occur in persons with dysfunctional RBFOX3 [2–5]. To address how *Rbfox3* is regulated during brain development, we determined the expression level of *Rbfox3* in wild-type (WT) mice from embryonic to adult stages (embryonic day 15.5, E15.5 to postnatal day 49, P49) in different regions of the brain (Fig 1a) with western blot analysis. Expression of *Rbfox3* was upregulated after birth and peaked at P21 in the cerebral cortex, striatum and cerebellum (Fig 1b, 1c and 1f, respectively), whereas the peak occurred at P14 in the hippocampus (Fig 1d) and amygdala (Fig 1e). Expression of *Rbfox3* was down-regulated in the adult brain in all regions examined (Fig 1). Our data suggest RBFOX3 could play an important role during brain development and maturation.

### Volumes from distinct brain regions were analyzed in WT and *Rbfox3*^*-/-*^ mice

**Fig 2:**
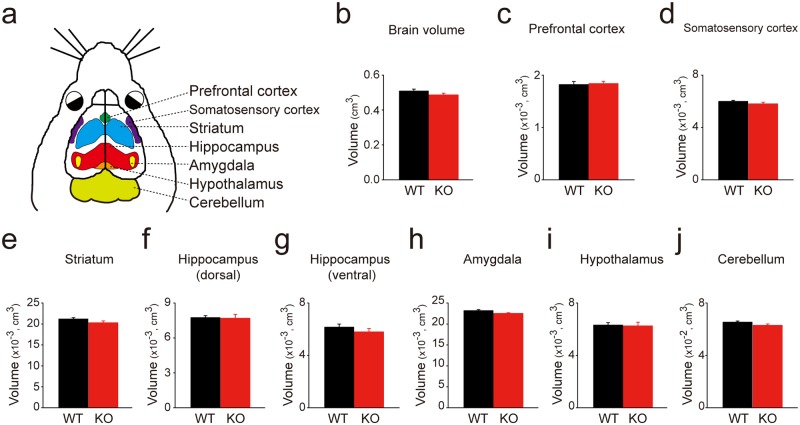
Volume measurements of distinct brain regions from wild-type and *Rbfox3*^*−/−*^ mice. (**a**) Schematic of adult mouse brain regions examined. Volumes of the whole brain (**b**), prefrontal cortex (**c**), somatosensory cortex (**d**), striatum (**e**), dorsal hippocampus (**f**), ventral hippocampus (**g**), amygdala (**h**), hypothalamus (**i**) and cerebellum (**j**) were measured in adult (postnatal day 56) wild-type (WT) and *Rbfox3*^*-/-*^ (KO) mice with MRI analysis. Student’s t-test, two-tailed, n = 6 per group.

*Rbfox3* is expressed developmentally in the brain and deletion of *Rbfox3* causes reduced brain weight regardless of gender [6], therefore RBFOX3 could affect brain structure during development. To address this question, we compared the volumes of different brain regions (Fig 2a) of WT and *Rbfox3*^*−/−*^ (KO) mice using MRI analysis (Fig 2). Brain volume was similar in WT mice and *Rbfox3*^*−/−*^ mice (Fig 2b). The volume was similar in both WT and *Rbfox3*^*−/−*^ mice in the prefrontal cortex (Fig 2c), somatosensory cortex (Fig 2d), striatum (Fig 2e), dorsal and ventral hippocampus (Fig 2f and 2g), hypothalamus (Fig 2i) and cerebellum (Fig 2j). However, we observed a trend of decreased volume in the amygdala (Fig 2h) in *Rbfox3*^*−/−*^ mice.

### *Rbfox3* deletion results in cold hyperalgesia and impaired cognitive abilities

**Fig 3:**
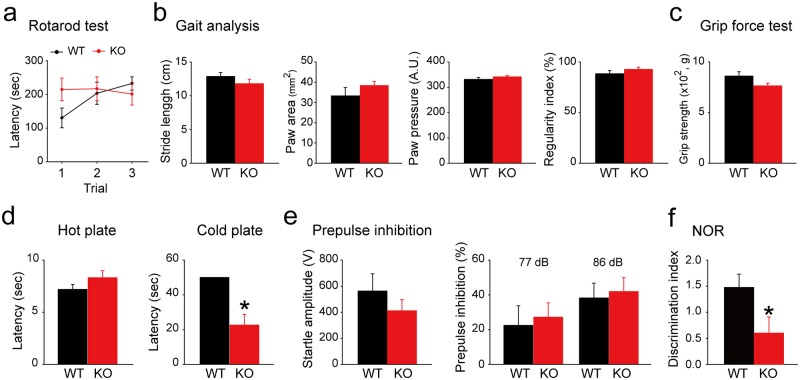
*Rbfox3*^*−/−*^ mice exhibited cold hyperalgesia and impaired cognitive ability compared to WT mice. (**a**) Rotarod test was performed for WT and *Rbfox3*^*−/−*^ (KO) mice. WT, n = 8, KO, n = 8. (**b**) Gait analysis was evaluated for WT and KO mice. WT, n = 12, KO, n = 9. (**c**) Grip force test was measured for WT and KO mice. WT, n = 12, KO, n = 9. (**d**) Hot/cold plate test was determined for WT and KO mice. All WT mice reached a 50 s cut-off time in the cold plate test; therefore, there is no error bar for WT mice. Mann-Whitney Rank Sum Test, *P < 0.05; WT, n = 8, KO, n = 8. (**e**) Evaluation of prepulse inhibition test for WT and KO mice. WT, n = 12, KO, n = 9. (**f**) Novel object recognition test was evaluated for WT and KO mice. Student’s t-test, two-tailed, *P < 0.05, WT, n = 11, KO, n = 13.

Because RBFOX2 is required for both cerebellar development and mature motor function [16] and RBFOX3 regulates alternative splicing and nonsense-mediated decay of *Rbfox2* [17], we investigated whether a deletion of *Rbfox3* could cause deficits in motor coordination. We performed a rotarod test, gait analysis and grip force test to evaluate the motor function and coordination of *Rbfox3*^*−/−*^ mice compared with WT mice (Fig 3). Motor coordination and grip force in *Rbfox3*^*−/−*^ mice was not significantly different from WT mice (Fig 3a, 3b and 3c). Deletion of *RBFOX3* was observed in persons with autism spectrum disorder [3]. Furthermore, persons with autism spectrum disorder experience sensory abnormalities related to sight, hearing, touch, smell and pain [18]. Therefore, we employed a hot/cold plate test and prepulse inhibition test to evaluate the nociceptive behavior and sensorimotor gating of *Rbfox3*^*−/−*^ mice compared to WT mice. When compared to WT mice, *Rbfox3*^*−/−*^ mice demonstrated cold hyperalgesia (Fig 3d), but there was no significant difference in the prepulse inhibition test (Fig 3e). Moreover, *Rbfox3*^*−/−*^ mice have learning deficits in the Water Maze test [6], therefore we wondered if these deficits could be reproduced in another learning paradigm, the novel object recognition test. Consistent with previous findings, *Rbfox3*^*−/−*^ mice exhibited learning deficits compared with WT mice (Fig 3f). Collectively, these data indicate that *Rbfox3*^*−/−*^ mice exhibit cold hyperalgesia and impaired cognitive abilities.

### *Rbfox3* deletion impairs adult neurogenesis

**Fig 4:**
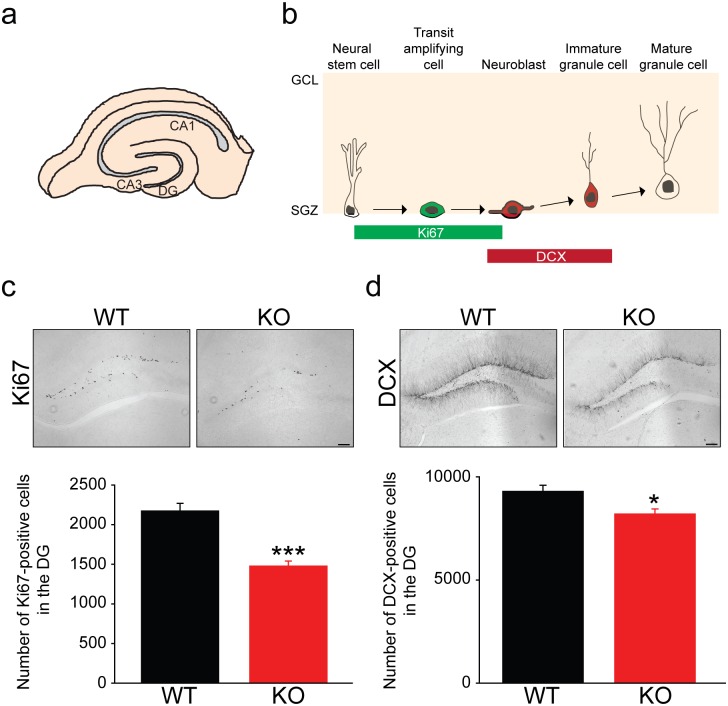
*Rbfox3* deletion causes deficits of neurogenesis. (**a**) Diagram of the location of the hippocampal dentate gyrus (DG). (**b**) Diagram showing different stages of postnatal neurogenesis in the hippocampal DG. Ki67 and DCX markers indicate distinct stages of neurogenesis. (**c**) Ki67 positive cells were quantified in the hippocampal DG of *Rbfox3* KO and WT mice. Student’s t-test, two-tailed, ***P < 0.001; WT, n = 42 sections, 6 sections/mice, 7 mice; KO, n = 36 sections, 6 sections/mice, 6 mice. (**d**) DCX positive cells were quantified in the hippocampal DG of *Rbfox3* KO and WT mice. Student’s t-test, two-tailed, *P < 0.05; WT, n = 54 sections, 6 sections/mice, 9 mice; KO, n = 48 sections, 6 sections/mice, 8 mice.

Since learning deficits were consistently identified in *Rbfox3*^*−/−*^ mice with two different paradigms of learning [6], we further investigated whether dysfunctional neurogenesis in the hippocampal DG (Fig 4a) could contribute to these deficits. Ki67, a marker for proliferation, and DCX, a marker for immature neurons, were used to estimate the level of ongoing neurogenesis [19, 20] (Fig 4b). We quantified both markers in the hippocampal DG of *Rbfox3*^*−/−*^ mice and WT counterparts and observed deficits in the number of Ki67- and DCX-positive cells (Fig 4c and 4d). In summary, these data indicate RBFOX3 plays an important role in adult neurogenesis. Its absence resulted in deficits in neurogenesis, which could contribute to the learning deficits seen in *Rbfox3*^*−/−*^ mice.

### *Rbfox3* deletion causes deficits in chemically induced long-term depression

**Fig 5:**
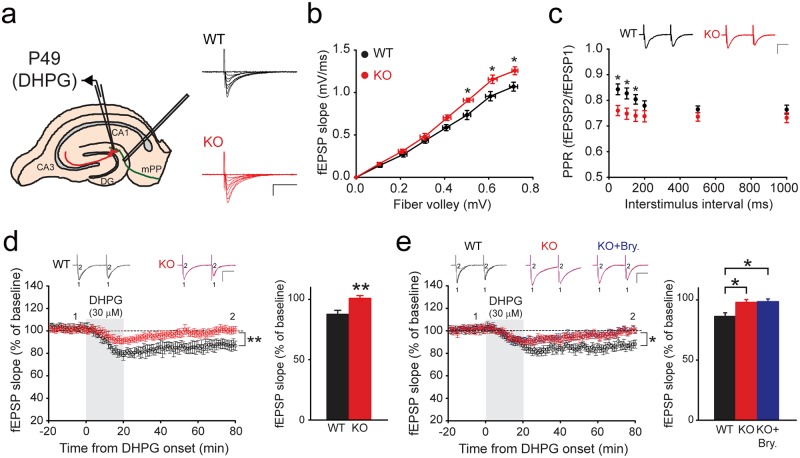
*Rbfox3*^*−/−*^ mice exhibited deficits of chemically induced long-term depression (LTD). (**a**) Schematic of recording configuration in hippocampal slices for LTD in the medial perforant path (mPP) of the dentate gyrus (DG). (**b**) Basal synaptic transmission from wild-type (WT) and *Rbfox3*^*−/−*^ (KO) mice was recorded and fiber volley amplitude plotted against the slope of field excitatory postsynaptic potential (fEPSP). WT, n = 7 slices, 3 mice; KO, n = 7 slices, 3 mice. Two-way repeated measures ANOVA with Holm-Sidak *post hoc* comparison, *P < 0.05. Representative traces with varying stimulus intensities from WT and KO mice are shown in (**a**). Scale bars represent 0.5 mV and 20 ms. (**c**) Analysis of paired-pulse ratio (PPR) at different interpulse intervals. Representative traces from WT and KO mice are from 50 ms inter-pulse intervals. Scale bars represent 0.5 mV and 20 ms (WT, n = 10 slices, 5 mice; KO, n = 10 slices, 5 mice). Two-way repeated measures ANOVA with Holm-Sidak *post hoc* comparison, *P < 0.05. **d**, Representative waveforms and averaged data of LTD following DHPG treatment. Bar graph shows lower LTD for KO than for WT mice. (WT, n = 10 slices, 5 mice; KO, n = 10 slices, 5 mice). Scale bars represent 20 ms and 0.5 mV. Two-way repeated measures ANOVA with Holm-Sidak *post hoc* comparison, **P < 0.01; Student’s *t*-test, two tailed, **P < 0.01. All data are the mean ± s.e.m. (**e**) Representative waveforms and averaged data of LTD following DHPG treatment with vehicle treatment in WT mice and with bryostatin-1 (Bry) or vehicle treatment in KO mice. Bar graph shows LTD in wild-type mice with vehicle treatment (black bar), knockout mice with vehicle treatment (red bar) and knockout mice with bryostatin-1 (Bry) (blue bar). WT, n = 6 slices, 3 mice; KO, n = 6 slices, 3 mice. Scale bars represent 20 ms and 0.5 mV. Two-way repeated measures ANOVA with Holm-Sidak *post hoc* comparison, *P < 0.05; One-way ANOVA with Holm-Sidak *post hoc* comparison, *P < 0.05. All data are the mean ± s.e.m.

Long-term depression (LTD) is a marker of synaptic plasticity and learning and memory. It is also a reliable indicator of learning inflexibility [21]. Activation of metabotropic glutamate receptors (mGluRs) by the agonist 3,5-dihydroxyphenlyglycine (DHPG) induced LTD (Fig 5a). We observed increased synaptic transmission (Fig 5b) and decreased paired-pulse depression (Fig 5c) in the hippocampal DG of *Rbfox3*^*−/−*^ mice compared with WT controls. More importantly, we observed deficits of chemically induced long-term depression (DHPG-LTD) in *Rbfox3*^*-/-*^ mice (Fig 5d), consistent with previous findings of electrically induced long-term depression (LFS-LTD) in *Rbfox3*^*-/-*^ mice[6], although both types of LTD use different receptors and upstream mechanisms[22]. Activation of protein kinase C (PKC) is necessary for induction of LTD [23–25]. However, LTD deficits in *Rbfox3*^*-/-*^ mice could not be rescued by applying the protein kinase C agonist, bryostatin-1 (Fig 5e). Our data suggest that deficits in LTD could be a reliable synaptic plasticity indicator of learning deficits in *Rbfox3*^*−/−*^ mice. PKC does not contribute to the deficits of LTD in *Rbfox3*^*−/−*^ mice.

### *Rbfox3* deletion causes abnormal exon usage of synapse-related genes and increases excitatory synaptic number

**Fig 6:**
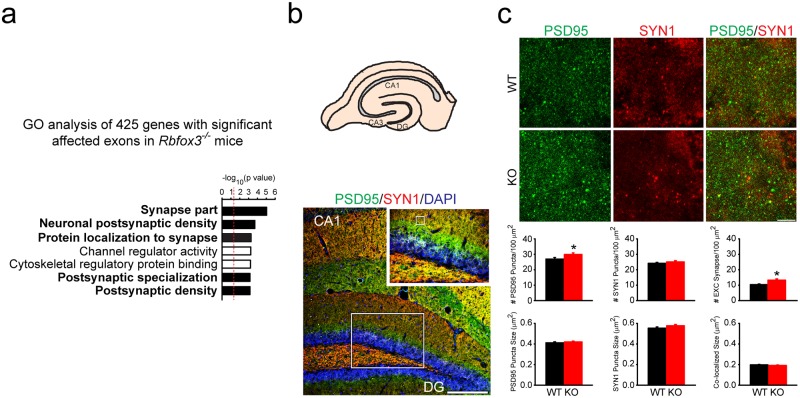
RBFOX3 regulates exon usage of synaptic related genes and *Rbfox3* deletion causes increased numbers of excitatory synapses. (**a**) GO analysis was performed for genes with affected exons. (**b**) Schematic of hippocampal dentate gyrus (DG) (top). Immunofluorescence staining of the DG (bottom); small box inside the large box indicates the area in which synapses were counted. Scale bar = 200 μm. Scale bar = 50 μm for the insert figure. (**c**) Top: Immunofluorescent staining was used to localize excitatory synapses with the postsynaptic marker (PSD95, green) and the presynaptic marker (SYN1, red) in the DG. Bottom: Quantification of the number and size of presynaptic and postsynaptic sites, and overlap. WT, n = 36 sections, 3 mice; KO, n = 36 sections, 3 mice. Scale bar = 5 μm. Student’s t-test, two tailed or Mann-Whitney Rank Sum Test, *P < 0.05. All data are the mean ± s.e.m.

Dysfunction of synapses could partly contribute to the increase in neurotransmission and abnormal synaptic plasticity we observed in *Rbfox3*^*−/−*^ mice [6]. Therefore, RBFOX3 could play an important role in synaptic function. To address this question, we first examined the exons affected by *Rbfox3* deletion with RNA-Seq. We found a significant relationship between genes with affected exons and synaptic genes (Fig 6a). Since it has been shown that RBFOX family proteins bind to the TGCATG motif around the affected exons [7, 26], we focused on affected exons with surrounding TGCATG motif. We observed that 101 out of 425 affected genes have surrounding TGCATG motif. The detailed information of affected genes is contained in S1 Table.

To further determine whether synaptic number could be affected by *Rbfox3* deletion, we counted the number of excitatory synapses in the hippocampal dentate gyrus of *Rbfox3*^*−/−*^ mice (Fig 6b and 6c). We examined the number of excitatory synapses in the dendrites of hippocampal dentate granule cells from *Rbfox3*^*−/−*^ mice and WT counterparts with the presynaptic marker (Synapsin 1, SYN1) and excitatory postsynaptic marker (PSD95). We found a significant increase in the number of excitatory synapses in *Rbfox3*^*−/−*^ mice in comparison to WT counterparts (Fig 6c). There was no change in size of the pre- or post- synaptic synapses in *Rbfox3*^*−/−*^ and wild-type mice (Fig 6c). Our results indicate that RBFOX3 regulates synaptic formation and function. In addition, increased excitatory synapses could partly contribute to the increased synaptic transmission in *Rbfox3*^*−/−*^ mice [6].

## Discussion

Dysfunctional RBFOX3 has been identified in various neurological disorders such as epilepsy, cognitive impairments, developmental delay and autism spectrum disorder. How exactly RBFOX3 causes its related brain disorders remains unclear and the role of RBFOX3 in the brain is largely unknown. Our data show that expression of *Rbfox3* differed developmentally for distinct brain regions and deletion of *Rbfox3* resulted in decreased neurogenesis. *Rbfox3*^*-/-*^ mice displayed cold hyperalgesia, impaired cognition and defective synaptic plasticity. Synapse-related genes are downstream targets of RBFOX3 and deletion of *Rbfox3* resulted in increased numbers of excitatory synapses.

RBFOX3 expression peaked at P14 in the hippocampus and amygdala and at P21 in the cerebral cortex, striatum and cerebellum. The peak of RBFOX3 expression is within the course of maturation for these distinct brain regions: at least 28 days in the hippocampus [27], amygdala [28], striatum [29], and cerebellum [30–32]; and at least 30 days in the cerebral cortex [33, 34]. This developmental match indicates the important role of RBOFX3 during brain development. In addition, this developmental expression of *Rbfox3* is consistent with previous findings for developmental expression patterns of *Rbfox3* in the whole brain [35]. In our current study, we not only detected cognitive deficits in *Rbfox3*^*-/-*^ mice with alternate behavioral tests of learning (novel object recognition test) but also examined other behavioral phenotypes related to changes in brain structures and autistic phenotypes. The observation of cold hyperalgesia in *Rbfox3*^*-/-*^ mice is indicative of a sensory abnormality, similar to abnormalities of pain experienced by persons with autism spectrum disorder [18]. Therefore, it will be important to conduct additional studies to determine how RBFOX3 contributes to this phenotype. Our previous finding showed a reduction in anxious behavior in *Rbfox3*^*-/-*^ mice [6]. The circuitry for fear and anxiety is associated with the amygdala [36], and although not significant, the observation of a trend toward a decreased volume in the amygdala is consistent with the anxiety behavior in *Rbfox3*^*-/-*^ mice. This suggests further investigations of the role of RBFOX3 in fear and anxiety should be explored.

Postnatal neurogenesis occurs throughout adult life in the subgranular layer of the hippocampal dentate gyrus and has been suggested to play a critical role in cognitive function [37]. Postnatal neurogenesis is a dynamic process, which is regulated by an enriched external environment [38–40] and the complex heterogeneous cellular environment [41]. The hippocampal subgranular layer contains constituent cell types, which include progenitor and dividing cells, immature granule cells, astrocytes and GABAergic interneurons [41]. Among these different cellular inputs, activity-dependent regulation by GABA neurotransmission has been shown to influence key steps of adult hippocampal neurogenesis [42, 43]. *Rbfox3* is only expressed in postmitotic neurons and our results show that decreased hippocampal neurogenesis occurs in *Rbfox3*^*−/−*^ mice. This suggests that RBFOX3 may contribute to the complex heterogeneous cellular environment for hippocampal neurogenesis through GABAergic interneurons. It would be interesting to further investigate whether RBFOX3 could affect other neurogenic areas such as the subventricular zone (SVZ), to verify whether the effect on neurogenesis through RBFOX3 is universal or brain region-specific. For instance, neuronal stem cells in the olfactory bulb originate from all rostrocaudal areas of the SVZ [44], therefore, it would be interesting to examine the SVZ of *Rbfox3*^*-/-*^ mice to determine if there is a deficit in neurogenesis and an alteration of olfactory phenotypes. However, because Ki67 is a marker for proliferative cells, and the variation of its number may include changes in the number of glial cells in addition to DCX-positive cells, we can not exclude the possibility that decreased neurogenesis in *Rbfox3*^*-/-*^ mice is accompanied by a decrease in gliogenesis. Unfortunately, examining gliogenesis was out of the scope of this study and was not investigated it further.

In our previous study [6], we showed that RBFOX3 regulates excitatory and inhibitory synaptic transmission, short-term and long-term synaptic plasticity, dendritic complexity, spine density as well as affecting synapse-related behavior. The current study demonstrates RBFOX3 also affects synaptic number and chemically-induced long-term plasticity (DHPG-LTD). Electrically and chemically-induced long-term depression use different receptors and upstream mechanisms [22], therefore, the findings in this study suggest RBFOX3 downstream targets could control both upstream molecules. Importantly, since abnormal synaptic transmission and plasticity were also observed in other brain regions such as hippocampal CA1 of *Rbfox3*^*-/-*^ mice [6], our observations of deficits of neurogenesis in the DG do not fully explain abnormal synaptic formation and plasticity in the DG of *Rbfox3*^*-/-*^ mice. Moreover, the functions of the downstream targets of RBFOX3 are predominantly synapse-related. The exon usage of those synapse-related genes was affected in *Rbfox3*^*-/-*^ mice, also indicating alterations in synapse-related genes and abnormal patterns of synaptic markers are not fully due to disrupted neurogenesis. Future studies should determine how RBFOX3 regulates its synapse-related targets and which targets can rescue phenotypes in *Rbfox3*^*-/-*^ mice.

Our earlier study observed increased spine density in the granule cells of *Rbfox3*^*-/-*^ mice [6], and although synapses are built on dendritic spines which are correlated with synapses, immature spines do not represent synapses [45, 46]. Therefore, to validate our previous findings, we examined the synaptic number with synaptic protein markers. There were significant increases in the number of synapses in *Rbfox3*^*-/-*^ mice, confirming our previous results, which strongly suggest RBFOX3 plays an important role in synaptic formation, maturation, function and plasticity. It has been shown that excitatory/inhibitory synaptic imbalance contributes to autistic features [47] and seizures [48]. Therefore, the increased number of excitatory synapses in *Rbfox3*^*-/-*^ mice could disturb the balance of excitatory and inhibition synaptic input, which could provide a potential explanation of autistic features and seizures observed in persons with disrupted RBFOX3.

Taken together, our results show that RBFOX3 contributes significantly to neural and synaptic maturation and function. Our observations of developmental expression of *Rbfox3* suggest RBFOX3 plays a crucial role during brain development. Our data also suggest RBFOX3 plays an important role in maintaining an environment for normal neurogenesis. Deficits of short-term and long-term plasticity in the hippocampal dentate gyrus in *Rbfox3*^*−/−*^ mice indicate RBFOX3 is essential for synaptic function. The combined data showing downstream targets of RBFOX3 are synapse-related and excitatory synapses increase in *Rbfox3*^*−/−*^ mice suggest RBFOX3 plays a role in the mediation of synaptic formation and function. It will be important to conduct additional studies to better quantify the trend towards a decrease in amygdala volume in *Rbfox3*^*-/-*^ mice. Future studies are needed to precisely determine how presynaptic and postsynaptic proteins function under the control of RBFOX3. Understanding these relationships could lead to the development of therapeutic agents, which could be used to target areas affected by dysfunctional RBFOX3 in persons with RBFOX3 neurodevelopmental brain disorders.

## Supporting Information

S1 Table Summary of exon usage and GO analysis in *Rbfox3*^*-/-*^ mice. (XLS)
